# Unique mechanical properties of nanostructured transparent MgAl_2_O_4_ ceramics

**DOI:** 10.1186/1556-276X-8-261

**Published:** 2013-06-01

**Authors:** Jie Zhang, Tiecheng Lu, Xianghui Chang, Nian Wei, Jianqi Qi

**Affiliations:** 1 School of Physics and Optoelectronic Engineering, Ludong University, Yantai 264025, People’s Republic of China; 2 Department of Physics and Key Laboratory for Radiation Physics and Technology of Ministry of Education, Sichuan University, Chengdu 610064, People’s Republic of China; 3 International Center for Material Physics, Chinese Academy of Sciences, Shenyang 110015, People’s Republic of China; 4 College of Physical Science and Technology, Southwest Jiaotong University, Chengdu 610031, People’s Republic of China

**Keywords:** Nanostructured transparent ceramic, Nanoindentation, Hardness, Elastic modulus

## Abstract

Nanoindentation tests were performed on nanostructured transparent magnesium aluminate (MgAl_2_O_4_) ceramics to determine their mechanical properties. These tests were carried out on samples at different applied loads ranging from 300 to 9,000 μN. The elastic recovery for nanostructured transparent MgAl_2_O_4_ ceramics at different applied loads was derived from the force-depth data. The results reveal a remarkable enhancement in plastic deformation as the applied load increases from 300 to 9,000 μN. After the nanoindetation tests, scanning probe microscope images show no cracking in nanostructured transparent MgAl_2_O_4_ ceramics, which confirms the absence of any cracks and fractures around the indentation. Interestingly, the flow of the material along the edges of indent impressions is clearly presented, which is attributed to the dislocation introduced. High-resolution transmission electron microscopy observation indicates the presence of dislocations along the grain boundary, suggesting that the generation and interaction of dislocations play an important role in the plastic deformation of nanostructured transparent ceramics. Finally, the experimentally measured hardness and Young’s modulus, as derived from the load–displacement data, are as high as 31.7 and 314 GPa, respectively.

## Background

Magnesium aluminate (MgAl_2_O_4_) spinel transparent ceramic has been considered as an important optical material due to its good mechanical properties and excellent transparency from visible light to infrared wavelength range [1]. However, it is well known that their intrinsic fracture toughness (premature failure due to brittle fracture) [2-4] limits their wide applications in severe environments. Therefore, there has been great interest in the investigation of ceramic materials with improved toughness [5-8]. In particular, it has been believed that nanostructured ceramics may have greatly improved mechanical properties when compared with their conventional large-grained counterparts [9].

In our previous work [10,11], we employed a novel technique to study the fabrication of nanostructured transparent ceramics. Moreover, we analyzed the transparency mechanism in these ceramics. Nanoindentation is a powerful technique widely employed to determine the mechanical properties of nanostructured materials [12,13]. However, during the past decades, nanoindentation test has been widely utilized to measure the mechanical properties of numerous materials including polycrystalline ceramics [14-16] rather than those of nanostructured transparent ceramics. In this paper, we use the nanoindentation technique to probe the mechanical properties of nanostructured transparent MgAl_2_O_4_ ceramics.

## Methods

High-purity nanostructured transparent MgAl_2_O_4_ ceramics with a grain size of approximately 40 nm, fabricated by high pressure-temperature sintering [10], were selected as the test material for the present study. The mechanical properties of ceramic samples were characterized using a nanoindentation technique (Hysitron Inc., Minneapolis, MN, USA). Nanoindentation experiments were carried out on the samples with a diamond Berkovich (three-sided pyramid) indenter. In all loading-unloading cycles, loading and unloading lasted 2 s, respectively, and with a pause at a maximum load (*P*_max_) of 5 s. More than 20 indentations were performed at each load. The employed load ranges from 300 to 9,000 μN. Hardness (*H*) and Young’s modulus (*E*_r_) were calculated based on the model of Oliver and Pharr approach [17]. The nanostructure of the samples was investigated by means of high-resolution transmission electron microscopy (HRTEM). The residual nanoindentation imprints were observed using a scanning probe microsope (SPM).

## Results and discussion

**Figure 1:**
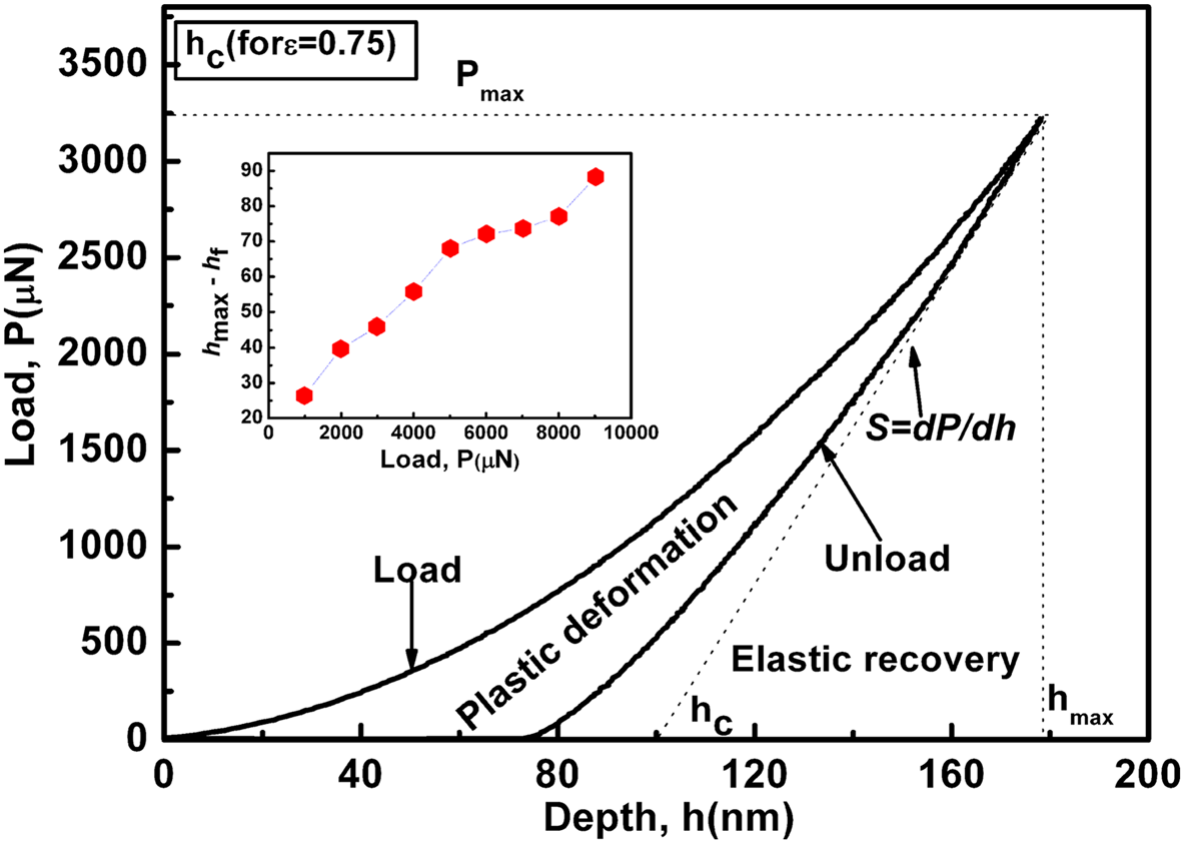
**Typical load-depth curve obtained from nanoindentation,*P***_**max **_**= 3,250 μN.** Inset shows the elastic recovery (*h*_max_ − *h*_f_) as a function of applied load.

Figure 1 shows a typical load-depth curve obtained through nanoindentation in the present study. The inset shows the difference between the total indentation depth at a maximum indented load (*h*_max_) and depth of residual impression upon unloading (*h*_f_), i.e., the elasticity recovery *h*_max_ − *h*_f_. Following the nanoindentation load-depth data, the *H* and *E*_r_ were determined [17]; these quantities can be derived using the following relations:

(1) S=dP/dh,

(2) $$h_{c}=h_{\max}-\epsilon •\frac{P_{\max}}{S},$$

(3) $$A=f\left(h_{c}\right)=24.56h_{c}^{2},$$

(4) $$H=P_{\max}/A,$$

(5) $$E_{r}=\frac{\sqrt{\pi }•S}{2•\beta •\sqrt{A}},$$

where *S* is the elastic constant stiffness defined as the slope of the upper portion of the unloading curve, as shown in Figure 1, *h*_c_ is the contact depth, *ϵ* is the strain (0.75 for the Berkovich indenter), *P*_max_ is the maximum applied load, *A* is the projected contact area at that load, *E*_r_ is the Young’s modulus, and *β* is the correction factor that depends on the geometry of the indenter (for the Berkovich tip, *β* is 1.034).

Also, we determined the elastic recovery (*h*_max_ − *h*_f_) for nanostructured transparent MgAl_2_O_4_ ceramics indented at different applied loads. The results showed that there was a higher degree of plastic deformation at a higher applied load, as shown in the inset of Figure 1.

The load-depth curve (Figure 1) is characterized by a substantial continuity, i.e., there are no large steps (pop-ins or pop-outs) observed in both loading and unloading. Figure 1 shows high elastic recovery (70.58%) and low plastic deformation (29.42%). However, when different loads were applied from 300 to 9,000 μN, it was observed that there was an appreciable increase in plastic deformation. In fact, from the present calculation of the depth before and after removal of the applied load, it was found that 57.72% of the total work done during the indentation is attributed to elastic deformation.

**Figure 2:**
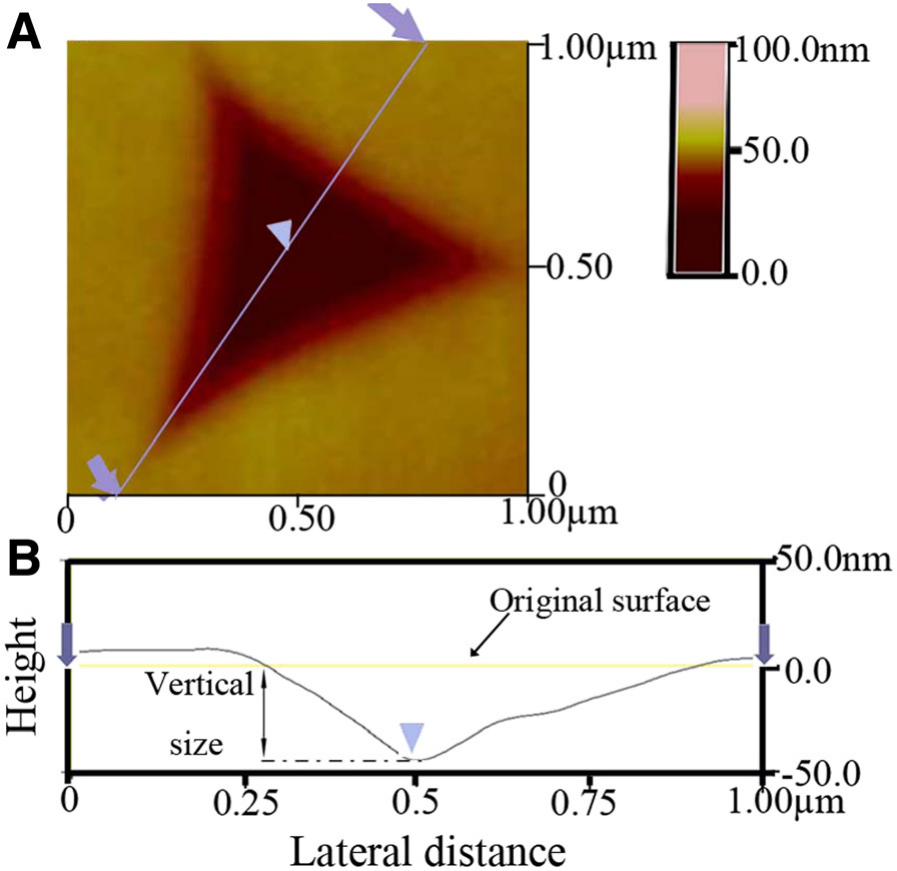
**SPM image and corresponding cross-sectional profile.** SPM image of an indented area (**A**) and the corresponding cross-sectional profile (**B**) along the bluish grey line in (**A**).

Images of the nanoindentation were captured by the SPM mode, as shown in Figure 2A, which confirms the absence of any cracks and fractures around the indented zone. Instead, the flow of the material along the edges of indent impressions can be clearly seen. This flow is substantiated via a line trace of SPM images along the diagonal section of the selected indent (bluish grey line in Figure 2A). The corresponding cross-sectional profiles are displayed in Figure 2B. Similar pile-ups around the indentation were observed in the nanocrystallization during the nanoindentation of a bulk amorphous metal alloy at room temperature, indicating the severity of plastic flow around this region during indentation [18]. Moreover, polycrystalline hydroxyapatite is reported to exhibit plasticity at higher temperature [19,20], but no plasticity has been reported at room temperature for nanostructured transparent ceramics. Furthermore, for ceramic materials, the plasticity is limited at low loads, and the influence of dislocation can be important [21,22]. Thus, the faceted pile-up region suggests that dislocations generated during the indentation are attributed to the residual strain of nanostructured transparent ceramics.

**Figure 3:**
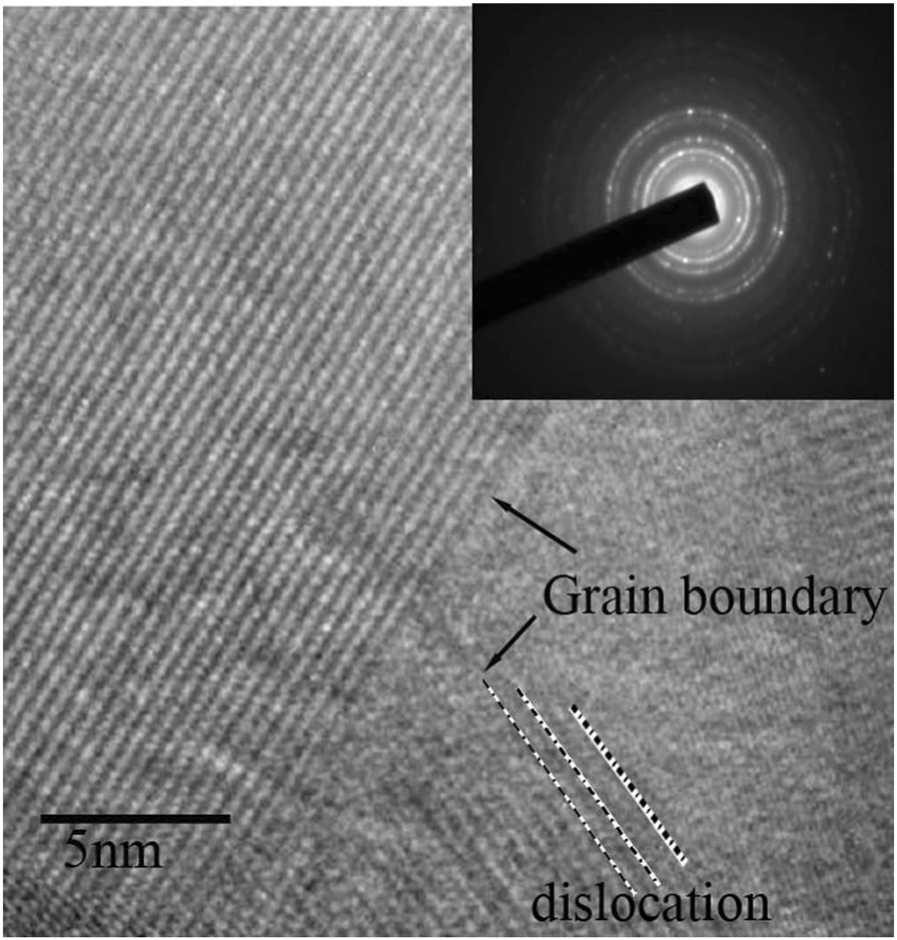
**HRTEM image of the nanostructured transparent MgAl**_**2**_**O**_**4 **_**ceramic.** Inset shows the selected area diffraction pattern.

In order to further investigate the mechanical properties of nanostructured transparent ceramics, we used HRTEM to examine the microstructures of the sample indented at 9,000 μN. The HRTEM image is shown in Figure 3. The inset in this figure is a selected area electron diffraction pattern of the indented sample, indicative of a magnesia-alumina spinel crystal structure. The left part of the HRTEM image reveals well-ordered atomic structures. However, there are dislocations close to the triangular grain boundary, suggesting that the generation, movement, and interaction of dislocations during the indentation play an important role in the plastic deformation as well as the resulting mechanical properties.

**Figure 4:**
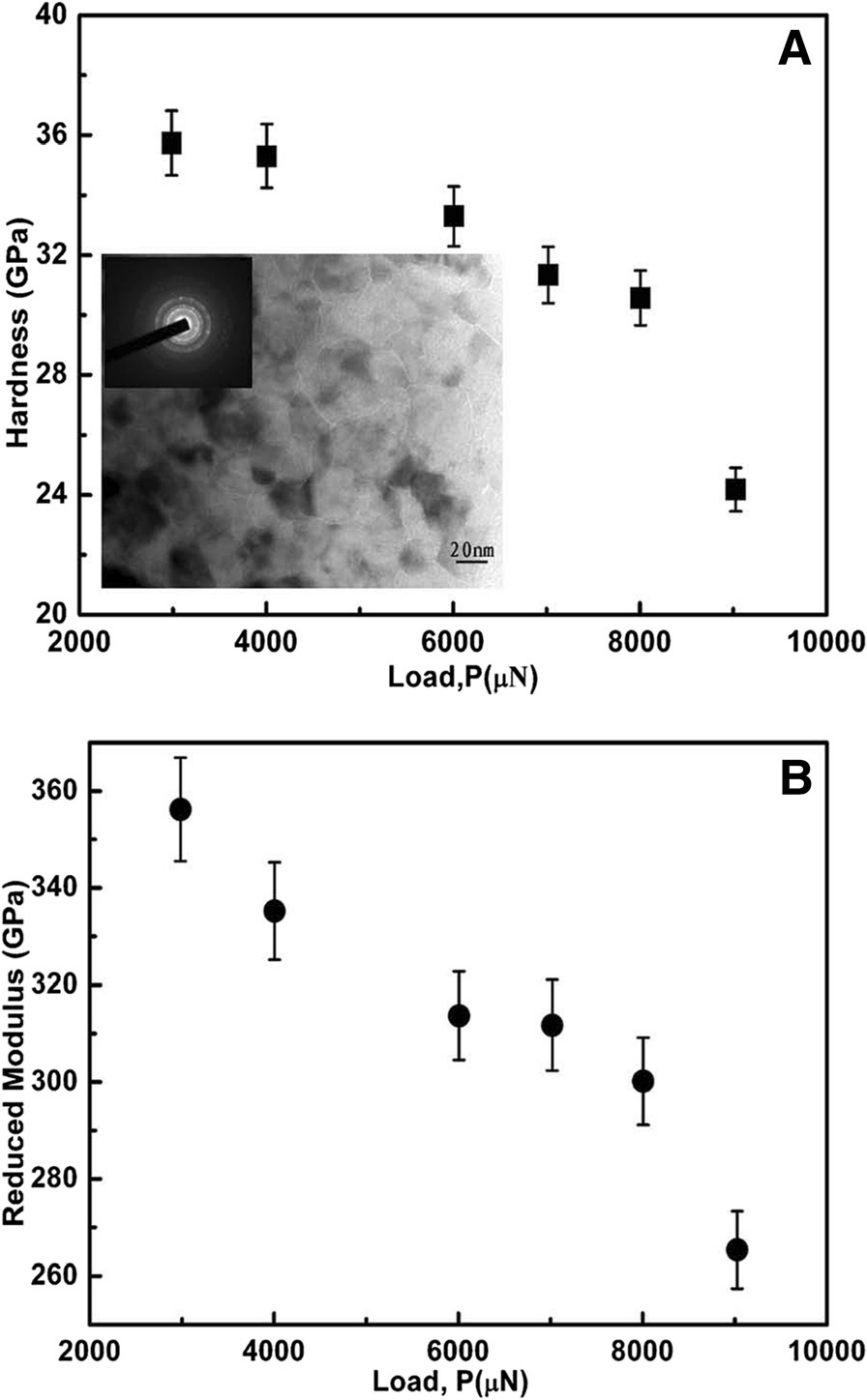
**Hardness (A) and Young’s modulus (B) as a function of applied load.** Inset shows TEM image of the sample.

Hardness and Young’s modulus of the nanostructured transparent MgAl_2_O_4_ ceramics are shown in Figure 4 as a function of the applied load. Both hardness and Young’s modulus decrease with increasing loads. Furthermore, it also indicates that there appears to be a larger decrease in the hardness than in the Young’s modulus with increasing load. These phenomena have been attributed to the well-known indentation size effect. Gong et al. [14] studied an alumina ceramic by nanoindentation testing and found that more cracks were generated at higher loads. However, the absence of cracks in the vicinity of the indented zone (Figure 2) suggests that it should not be reasonable to explain the load-dependent mechanical properties of our nanostructured transparent ceramics only by the indentation size effect. Dislocation activity, as evidenced in Figure 3, compared to HRTEM images of the sample at atmospheric pressure [11] should be considered as an important factor that can influence the mechanical properties of nanostructured transparent ceramics. A more detailed study is clearly needed to understand how the dislocation activity influences the mechanical properties.

It has been observed that the hardness and modulus of ceramic materials with a smaller grain size have stronger load dependence than those with a larger grain size [23]. However, Young’s modulus is independent of the applied load when the load is above 10 mN [21]. Moreover, the contact depths in nanostructured samples indented at the lowest peak loads are already equal to or larger than the average grain size, and thus, Young’s modulus does not show any variation with increasing applied load [24]. In order to compare the hardness and modulus of our nanostructured transparent ceramics with those of conventional large-grained ceramics, we averaged the hardness and modulus data shown in Figure 4. The average hardness and modulus are 31.7 and 314 GPa, respectively. Our average hardness is approximately twice that of large-grained (100 to 200 μm) MgAl_2_O_4_[25]. This is understandable since the well-known Hall–Petch relationship predicts that a material with a smaller grain size should be harder than the same material with a larger grain size. Both the average modulus (314 GPa) and the modulus (265 GPa) measured at the maximum load (9,000 μN) are comparable to the Young’s modulus (277 GPa) of large-grained (100 to 200 μm) MgAl_2_O_4_[25]. This is also reasonable since it has been predicted that [26] the difference in Young’s modulus between porosity-free nanostructured materials with a grain size larger than 10 nm and conventional large-grained materials should be within approximately 5%.

## Conclusion

In summary, the deformation behavior and the mechanical properties (hardness and Young’s modulus) of the nanostructured transparent MgAl_2_O_4_ ceramics have been determined by nanoindentation tests. The degree of plastic deformation increases with increasing applied loads. After the indentation test, scanning probe microscope image shows no cracking, whereas high-resolution TEM image shows the evidence of dislocation activity in nanostructured transparent MgAl_2_O_4_ ceramics. The measured hardness is much higher than that of conventional large-grained MgAl_2_O_4_ ceramics, which should be of considerable interest to the fields of materials science and condensed matter.

## Competing interests

The authors declare that they have no competing interests.

## Authors’ contributions

JZ carried out the sample preparation, analyzed SPM, and participated on the nanoindentation analysis and paper corrections. TL analyzed the microstructures, evaluated the hardness and modulus, and designed the study. XC analyzed the TEM and HRTEM. NW and JQ participated in the study coordination and paper correction. All authors read and approved the final manuscript.
